# Towards a common template for neural reinforcement of finger individuation

**DOI:** 10.1038/s41598-020-80166-8

**Published:** 2021-01-13

**Authors:** Justin Kilmarx, Ethan Oblak, James Sulzer, Jarrod Lewis-Peacock

**Affiliations:** 1 Department of Mechanical Engineering, The University of Texas at Austin, 2501 Wichita St, Austin, TX 78712 USA; 2 Department of Psychology, The University of Texas at Austin, 108 E Dean Keeton St, Austin, TX 78712 USA

**Keywords:** Neurological disorders, Stroke, Computer science, Biomedical engineering

## Abstract

The inability to individuate finger movements is a common impairment following stroke. Conventional physical therapy ignores underlying neural changes with recovery, leaving it unclear why sensorimotor function often remains impaired. Functional MRI neurofeedback can monitor neural activity and reinforce it towards a healthy template to restore function. However, identifying an individualized training template may not be possible depending on the severity of impairment. In this study, we investigated the use of functional alignment of brain data across healthy participants to create an idealized neural template to be used as a training target for new participants. We employed multi-voxel pattern analyses to assess the prediction accuracy and robustness to missing data of pre-trained functional templates corresponding to individual finger presses. We found a significant improvement in classification accuracy (*p* < 0.001) of individual finger presses when group data was aligned based on function (88%) rather than anatomy (46%). Importantly, we found no significant drop in performance when aligning a new participant to a pre-established template as compared to including this new participant in the creation of a new template. These results indicate that functionally aligned templates could provide an effective surrogate training target for patients following neurological injury.

## Introduction

Deficiencies in the strength and control of individual fingers are commonly seen post-stroke^1,2^. These impairments of fine motor control manifest as an inability to move a single finger while keeping the other fingers stationary, impairing an individual’s ability to complete activities of daily living^3,4^. Conventional physical therapy of the affected limb often provides limited success at restoring fine-motor function^5–7^. It is difficult to determine why sensorimotor function remains impaired following stroke without a detailed understanding of how the brain recovers after injury. Even with this information, we currently lack the proper tools to intervene at the neural level. Evidence suggests that restoration of healthy brain patterns in the ipsilesional hemisphere, especially in those with milder injuries, is most closely associated with recovery and thus could be used as a model for neural interventions^8–11^. In contrast to using a neural target, conventional therapy that targets behavior ignores underlying neural changes. This could result in unintended maladaptive neuroplasticity arising from activation of ipsilateral motor projections or competitive interactions which may limit functional gains^12,13^. In order to advance physical therapy to the level of the injury, particularly in an application as granular as the fingers, brain imaging technology is required to guide neuroplasticity.

Functional magnetic resonance imaging (fMRI) neurofeedback has been proposed as a method to both monitor and target the affected circuitry in the brain^14^. In this procedure, participants are presented with a visual, auditory, and/or haptic representation of their neural activity in real time for the purpose of self-modulation^15^. Neurofeedback relies on the principles of operant conditioning where the provided feedback serves as a reinforcement for a desired behavior^14^. In a seminal work by Shibata et al.^16^, fMRI neurofeedback was shown to influence neural activity in the visual cortex. Participants in this study learned to self-modulate an autologous target pattern of brain activity corresponding to a visual grating without stimulus presentation or explicit instruction. This ability to learn to self-modulate neural activity resulted in improved behavioral performance for the target. Neurofeedback has also been explored in therapeutic fields such as the treatment of schizophrenia^17,18^, depression^19,20^, Parkinson’s disease^21^, attention disorders^22,23^, chronic pain^24^, and addiction^25,26^. There have also been several studies investigating neurofeedback as a treatment for motor recovery after stroke^27–29^, although none of these have attempted to target fine-motor control of individual fingers.

Previous research has shown that individuated finger movement elicits distinct patterns of brain activity that are measurable by multi-voxel pattern analysis (MVPA) of fMRI data from the sensorimotor cortex^30,31^. Strengthening these unique activity patterns has also been linked to improved motor control, such as improved reaction time and movement accuracy, in healthy individuals^32–34^. In a recent study from our group, finger preference during a motor task was altered after healthy participants were trained to endogenously shift their finger representations during fMRI neurofeedback training^35^. In a typical neurofeedback experiment, the neural target would be derived anatomically or functionally from the participant^36^. After stroke, however, altered neural representations possibly resulting from maladaptive neuroplasticity and an inability to individuate finger movements^3,4^ would make autologous neural training targets untenable. Therefore, it is necessary to develop a template of fMRI brain activity from healthy participants to serve as a surrogate training target.

**Figure 1:**
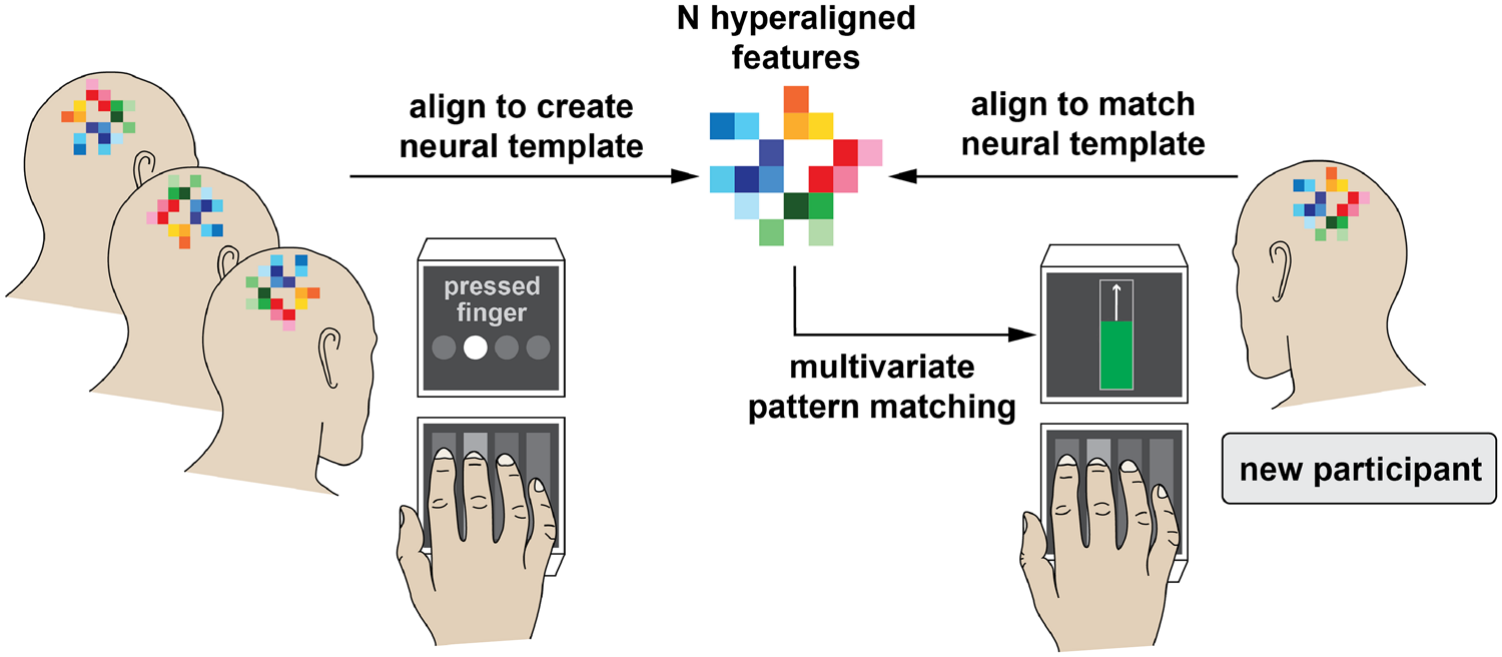
Overview of functional-based alignment. Neural patterns are rotated from subjects’ native space into a common template space. This template can then serve as a surrogate neurofeedback training target for a new participant who is unable to create an autologous target due to neurological injury.

Creating an fMRI template requires compounding data from multiple participants to generate a representative brain activation pattern for a given movement as outlined in Fig. 1. Anatomical alignment based on brain landmarks is perhaps the most conventional method of between-subject analysis; however, this alignment procedure is often imperfect due to topographic variability between individuals^37–40^ and produces lackluster classification performance^41–43^ that is likely insufficient for neurofeedback training^44^. More recently, a new technique known as *hyperalignment* has emerged as a method for improving across-subject fMRI analysis^42,45,46^. Hyperalignment works by applying the Procrustean transformation^47^ to functionally align voxel activity across individuals into a common model space. By compounding data across multiple individuals, a hyperaligned model benefits from a greater pool of training data and can often outperform within-subject methods^41,43,48^.

One other advantage of hyperalignment is its ability to generalize to new data^48^. Recently, Taschereau-Dumouchel et al.^43^ used hyperalignment to infer the neural representation for feared animals (e.g., a snake) in a given participant with a snake phobia based only on data from other participants. This allowed the researchers to obtain an accurate neural target of the feared animal without actually submitting the participant to the fearful stimulus. A similar strategy could be used for stroke patients who are unable to produce representations for a given finger due to neurological injury. For instance, if a patient is unable to produce an individuated movement in one finger, it may be possible to remove the impaired finger from the hyperalignment calculations and infer its representations from the data of healthy participants.

**Figure 2:**
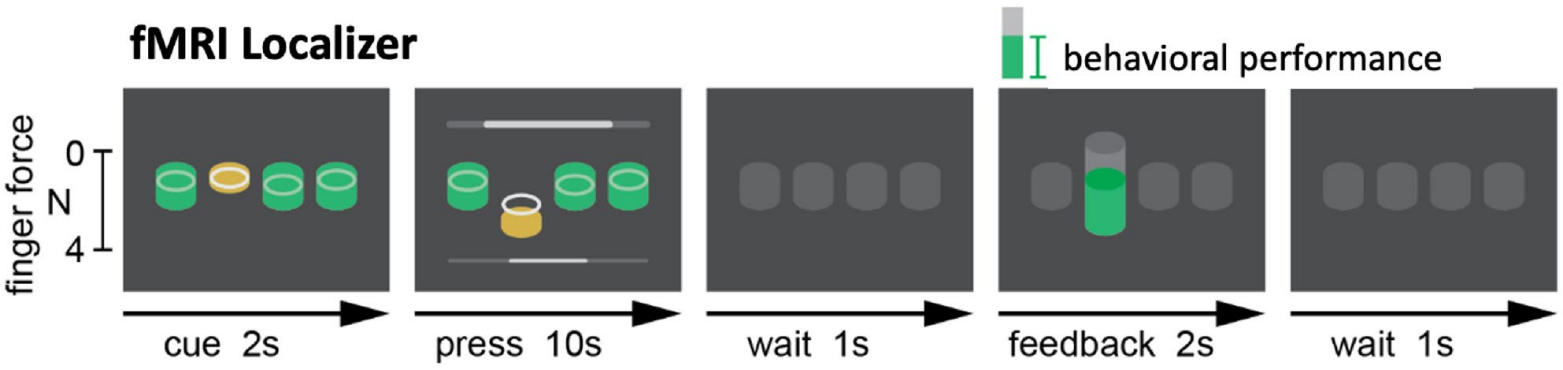
Experimental task protocol. Participants were cued to press one finger for 10 s while maintaining a constant force on the non-cued fingers. Feedback representing the number of correct presses was presented at the end of each trial. This process was repeated for 8 runs of 20 trials each.

In this study, we sought to investigate the use of hyperalignment to create a neural template of individual finger presses from healthy individuals. Such a template could be used in an fMRI neurofeedback intervention for patients with fine motor deficiencies and who are unable to provide a personalized training target. We conducted an offline analysis to validate this method on previously gathered data from N = 17 healthy participants in a single session to identify unique fMRI activity patterns in sensorimotor cortex for each of the four fingers (index, middle, ring, and little) of the right hand^35,44^. During this session, participants were asked to press one cued finger while maintaining a constant force with the non-cued fingers (Fig. 2). The fMRI data was used to train three separate pattern classifiers to discriminate between the four fingers: a within-subject classifier trained in a participant’s native space, a between-subject classifier trained on anatomically aligned data in MNI152 (Montreal Neurological Institute) standard-space, and a between-subject classifier trained on hyperaligned data from other participants. Because our data was collected from healthy individuals with high individuation abilities, the results of the within-subject classifier serve as a theoretical maximum. The between-subject classifier trained on anatomically aligned data serves as the main point of reference for which we evaluate the performance of hyperalignment.

We hypothesized that hyperalignment would provide better classification results than the traditional anatomical alignment. We also tested the model’s ability to generalize to finger representations that have been removed during alignment. Finally, we addressed several important methodological questions about the implementation of hyperalignment: the effect of subject order on hyperalignment, the effect of aligning a new individual to a pre-established common model (compared to creating a new common model informed by all participants), and how much data is required to accurately align a new participant to a common model space. These results will address whether creating a common neural template from healthy individuals can facilitate fMRI neurofeedback rehabilitation for patients with neurological impairment.

## Results

### Hyperalignment provides improved between-subject classification

**Figure 3:**
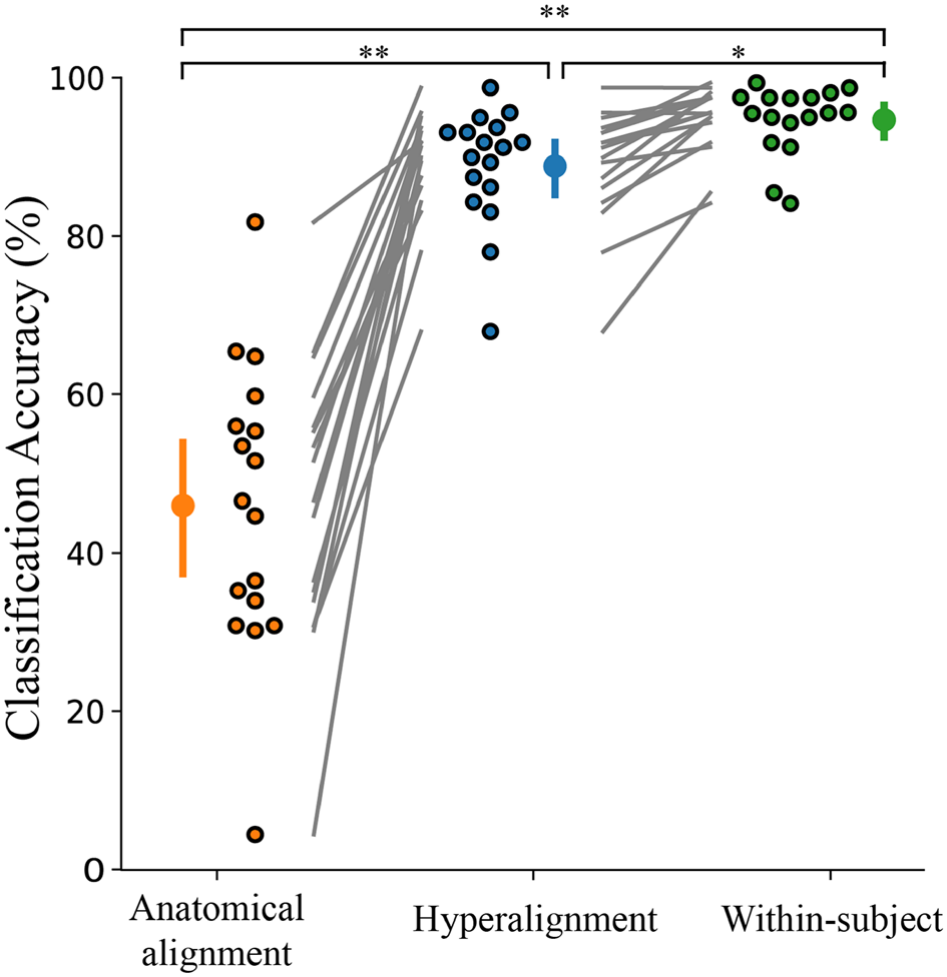
Hyperalignment provides better between-subject classification accuracy (88.8%) than conventional anatomical alignment (46.0%). Within-subject classification (94.7%) is also included for comparison. Individual participants are presented beside the mean as circles with a black outline. Error bars indicate a 95% confidence interval. *p < 0.05, **p < 0.001.

We first sought to compare the prediction accuracies between the three classifiers trained on hyperaligned, anatomically aligned, and within-subject data. The results are presented below in Fig. 3. A one-way ANOVA revealed a statistically significant difference between groups (F(2,27.1) = 57.8, *p* < 0.001). The between-subject classification model trained on hyperaligned data performed significantly better than the classifier trained on anatomically aligned data (88.8% and 46.0%, respectively; Games-Howell post-hoc test, *p* < 0.001). The within-subject model trained on the participants’ own data yielded 94.7% classification accuracy. As an additional test, a within-subject analysis on the participants’ own data after anatomical alignment into MNI space provided 94.0% classification accuracy. This verified that warping participants’ data into MNI space did not significantly affect classification accuracy (t(16) = 0.871, *p* = 0.396).

### Physical and representational overlap between neighboring fingers

**Figure 4:**
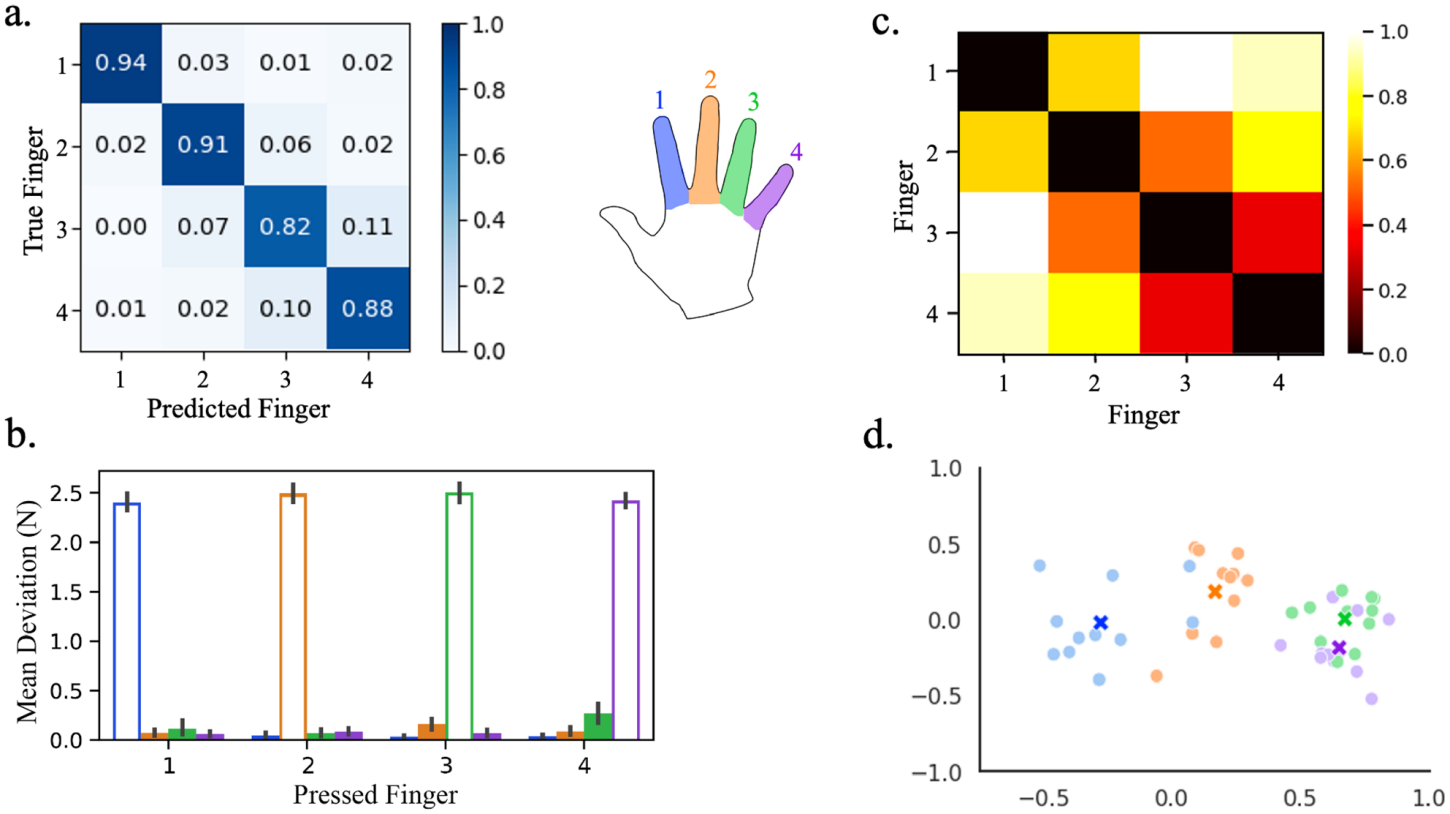
Representational overlap between neighboring digits contribute to increased misclassification of finger presses. (**a**) Confusion matrix of classifier predictions trained on hyperaligned data. (**b**) Mean deviation from baseline force of the uninstructed fingers (filled bars) compared to the mean press force of each instructed finger (hollow bars). Error bars indicate a 95% confidence interval. (**c**) Average representational dissimilarity matrix of neural patterns associated with finger presses. (**d**) Two-dimensional projection of the representation structure using multidimensional scaling. Dissimilarity is reflected by the distance in the 2 dimensions. The mean representation across participants in panel **d** is shown as an ‘X’. Individual digits are represented by different colors and numbers (see key in center). Data is only shown from the 11 participants from Oblak et al.^35^ whose force data was collected.

A deeper look into the predictions of the classifier trained on hyperaligned data in Fig. 4a indicates increased misclassification between the ring and neighboring fingers compared to the other finger pairs. To better understand how this classifier was influenced by the uninstructed fingers, we analyzed the degree of physical overlap, measured by mean deviation from baseline force^49^, and representational overlap, measured by representational similarity between neural patterns^50^. Only information from the 11 participants from Oblak et al.^35^ whose responses were collected with the force keyboard were included in these analyses.

The force data presented in Fig. 4b shows the mean deviation from baseline force of the uninstructed fingers compared to the mean press force of the instructed finger. The mean deviation of each uninstructed finger was inappreciable at a target press of around 2.5 N, which would be expected at such a low level of target force^49^. However, the results of the representational similarity analysis revealed greater representational overlap in neighboring digits, particularly between the ring and little finger pair (**F**ig. 4c,d). Therefore, these overlapping representations are responsible for the increased classifier confusion between neighboring digits.

### Hyperaligned common model generalizes to novel fingers

To validate the common model’s ability to generalize to new data, we removed the fMRI data from select fingers prior to feature selection and hyperalignment parameter calculations in all participants. Hyperalignment was performed on the remaining fingers to create the common model space and obtain a transformation matrix for each individual. These transformation matrices were then applied to the dataset containing fMRI data from all fingers. This provided a versatile source of inference of the removed finger representations for the test subject.

The hyperaligned model yielded an overall classification accuracy of 80.6% when one finger was removed during hyperalignment. Furthermore, the hyperaligned model was able to infer the representations of the removed finger from the surrogate participants and achieve above-chance classification (mean 69.3%, chance level 25%) for the removed finger (one-way ANOVA, F(1,75.2) = 384, *p* < 0.001). When two fingers were removed during hyperalignment, the classifier still provided above-chance classification of all fingers (mean 66.5%, F(1,113) = 1035, *p* < 0.001) and for the two removed fingers (mean 56.9%, F(1,204) = 428, *p* < 0.001). When three fingers were removed during hyperalignment, the overall classification accuracy dropped to 35.6%. While still significantly above chance (F(1,82.8) = 55.9, *p* < 0.001), this would likely not provide an accurate template.

**Figure 5:**
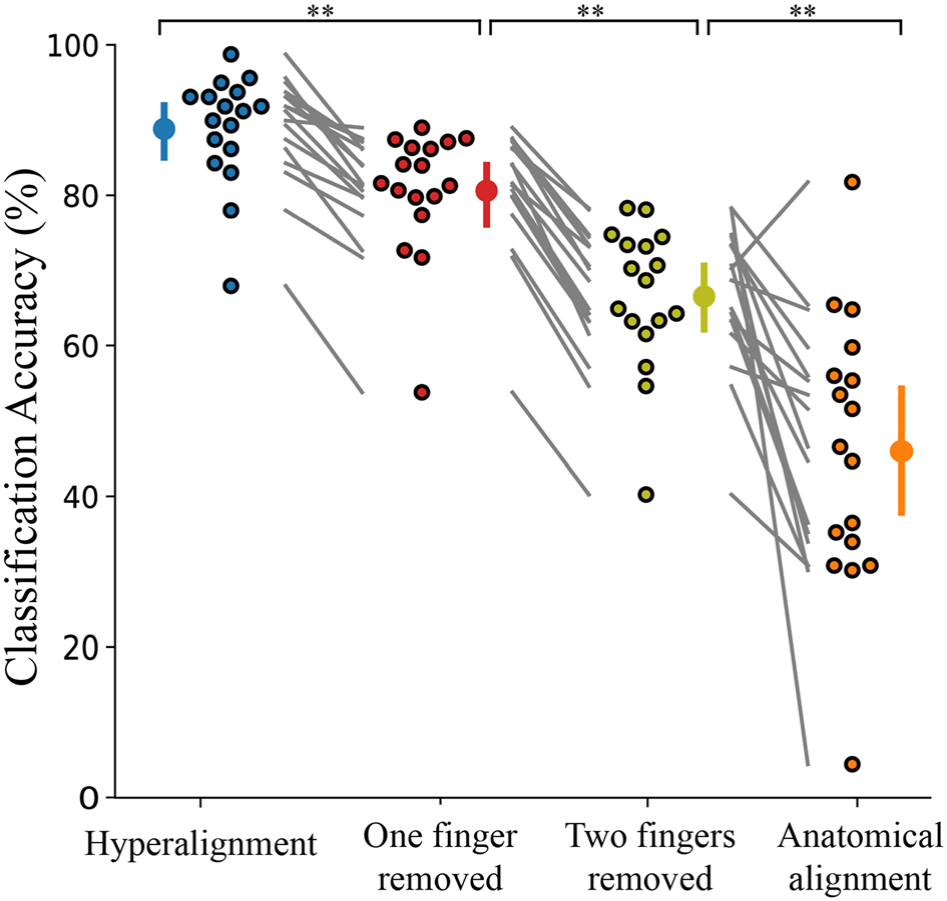
Overall classification accuracy for all four fingers when all fingers are included in hyperalignment parameter calculations (88.8%), when one finger is removed during hyperalignment (80.6%), when two fingers are removed during hyperalignment (66.5%), and anatomical alignment with all fingers included (46.0%). Individual participants are presented beside the mean as circles with a black outline. Error bars indicate a 95% confidence interval. **p < 0.001.

Figure 5 shows the overall classification accuracies when one and two fingers were dropped during hyperalignment along with the prediction accuracy when all fingers were included in the hyperalignment procedure and during anatomical alignment for comparison. There was a statistically significant difference between groups (F(3,194) = 92.0, *p* < 0.001). While the model suffered a decrease in classification accuracy when one or two fingers were removed compared when all fingers where included during common model generation, it still performed better than baseline anatomical alignment with all fingers included (Games-Howell post-hoc analysis, *p* < 0.001).

### Hyperaligned common model generalizes to new individuals

**Figure 6:**
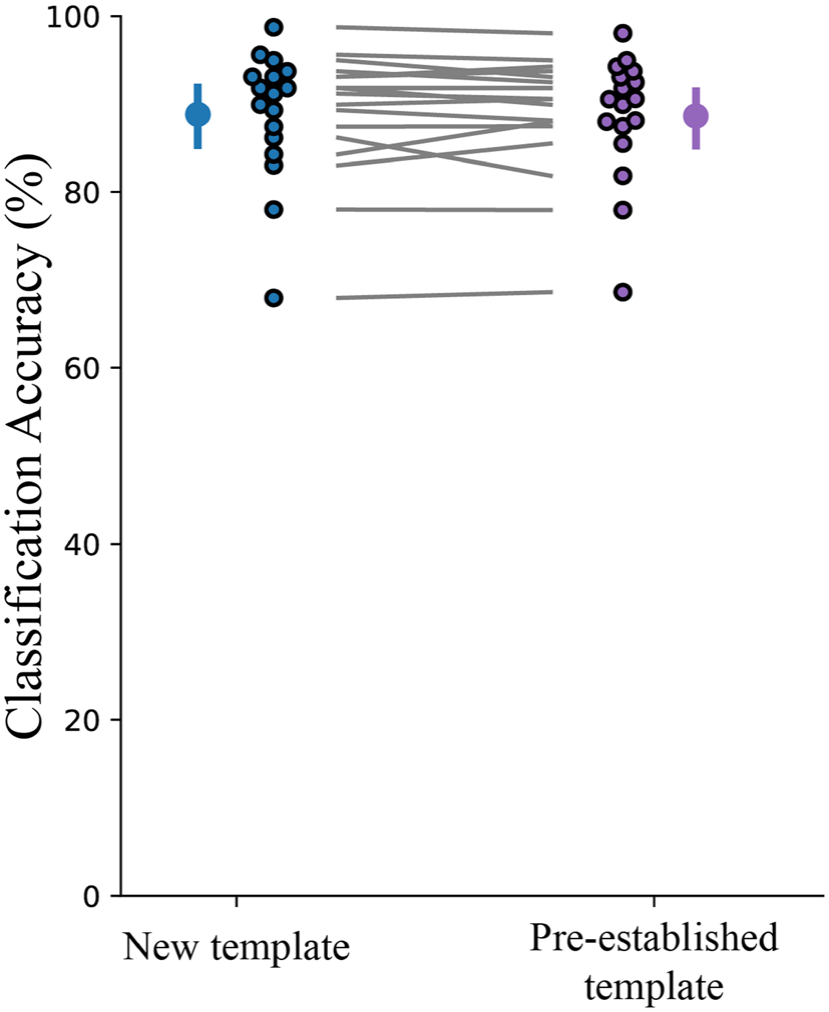
Decoding effects of using all participants to create a template space versus hyperaligning a new participant to a pre-established template. Individual participants are presented beside the mean as circles with a black outline. Error bars indicate a 95% confidence interval.

To further validate the effectiveness of a surrogate training target, we evaluated the classification accuracy when a new individual was brought into alignment with a pre-established common model space. This would be the most likely scenario in a neurofeedback paradigm where a template is used as a training target for a new participant. Previous studies have indicated that classification accuracy decreased when mapping a new participant onto a previously created common model^41^. However, we saw no significant difference between including a new participant in the creation of a new common model vs. aligning a new participant to a pre-established common model (Fig. 6; t(16) = 0.433, *p* = 0.67). This difference of findings may be attributed to a lower degree of inter-subject variability in our experiment compared to other studies. Further deliberation is included in the Discussion.

### Effect of subject order on common model generation

**Figure 7:**
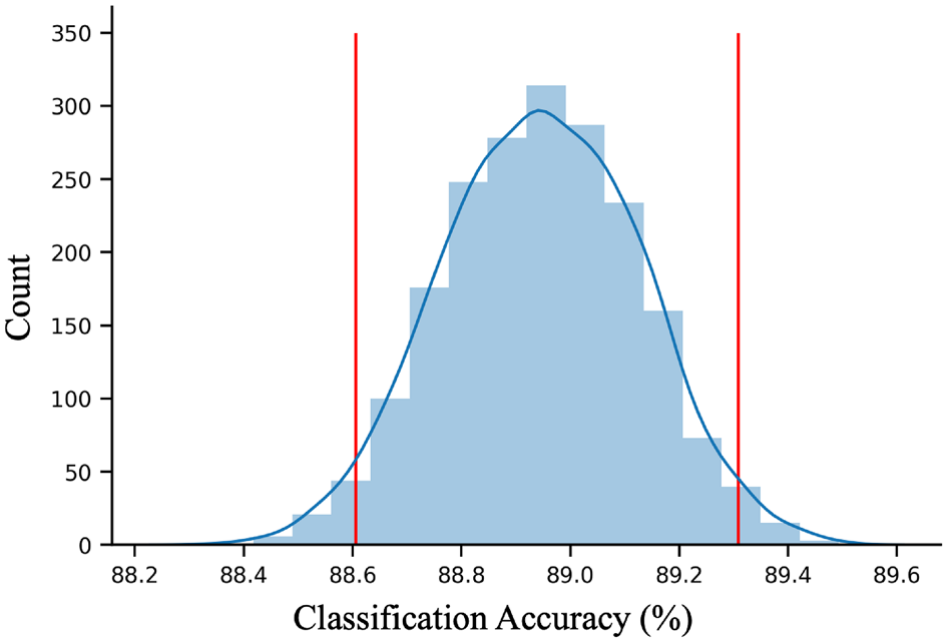
Histogram of mean classification accuracy for hyperalignment of 2000 permutations of random subject order. Red vertical bars demarcate the 95% confidence interval.

It has also been suggested that the order in which participants enter the hyperalignment process affects the overall model’s performance^41^. To assess the effects of subject order, we repeated our hyperalignment classification analysis over 2000 permutations of random subject orders. Figure 7 presents the distribution of mean between-subject classification accuracies as a histogram with a mean classification accuracy of 88.95% (95% confidence interval, 88.61–89.31%). These results indicate a very minimal effect of subject order on overall model performance.

### How much data should be collected for a new participant?

**Figure 8:**
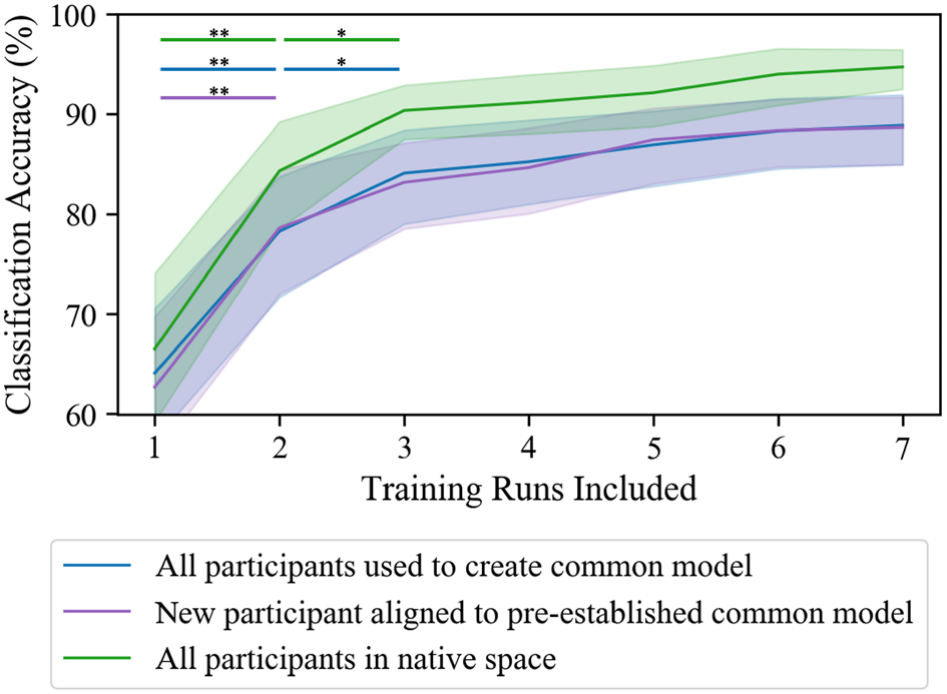
Number of runs needed to hyperalign individuals and train an accurate classifier. The results indicate that at least two runs are necessary to accurately align a new participant to a pre-established common model. At least three runs are necessary for the between-subject classifier using all participants to create the common model and the within-subject classifier trained in a subject’s native space. Shaded outline indicates 95% confidence interval. *p < 0.05, **p < 0.001.

The final question we addressed was how much data needs to be collected from a new participant to accurately hyperalign them to a pre-established common model. Figure 8 shows the classifier’s performance as the number of runs included in hyperalignment calculations and classifier training was increased. A between-subject classifier using all participants to create a common model and a within-subject classifier trained on the same amount of data is also included for comparison. The results of a one-way repeated-measures ANOVA showed that there was a significant main effect of the number of runs included on prediction accuracy for the classifier where a new subject was aligned to a pre-established common model (F(1.88,16) = 59.3, *p* < 0.001), the classifier using all participants to create a common model (F(2.10,16) = 50.6, *p* < 0.001), and the classifier trained in a participant’s native space (F(1.46,16) = 51.3, *p* < 0.001). Tukey post hoc tests revealed that the inclusion of additional data significantly improved classification accuracy up to three runs for the classifier using all participants to create a common model and the classifier trained in a participant’s native space. The inclusion of additional data significantly improved classification accuracy only up to two runs for the classifier where a new subject was aligned to a pre-established common model.

## Discussion

The results of this study demonstrate the validity of using hyperalignment to construct a neural template of brain activity to be used as a surrogate training target in a neurofeedback experiment. Hyperalignment of the primary motor and somatosensory cortices across individuals provides a 42.8% improvement in classification accuracy of individual finger presses compared to anatomical alignment. While other studies have reported improved classification accuracy after hyperalignment compared to within-subject analysis^41,43,48^, participants in this study were able to elicit highly individuated finger movements which led to high within-subject classification. Due to this ceiling effect, our hyperaligned model was not able to outperform a within-subject model. However, we have shown that greater representational overlap between fingers contributes to increased classifier confusion. In patients with neurological injury, impairments in fine-motor control are accompanied by greater representational overlap which could substantially reduce a classifier’s prediction ability. This may make identifying an appropriate autologous neurofeedback training target impossible. For this reason, we recommend functional alignment to a healthy neural template. This would provide a larger pool of data to boost classification performance and provide a more precise training target.

We have also shown that hyperalignment is able to generalize to missing data consistent with the findings of Taschereau-Dumouchel et al.^43^. This would be advantageous for participants who have significant motor impairments and cannot produce distinct neural representations for each finger. The impaired finger could be removed during construction of the common model, and the data from the other fingers could be used to infer the representation of the removed digit. While there was a significant decrease in the predication accuracy of the removed finger itself, this functionally aligned classifier still outperformed a classifier trained on anatomically aligned data. Furthermore, the study conducted by Taschereau-Dumouchel et al.^43^ removed only one out of 40 different categories whereas our study has shown significant prediction abilities even after half of the data has been removed (two out of four categories).

In a recent study conducted by Al-Wasity et al.^41^, a similar approach of using a hyperaligned common model to predict imagined arm movements was significantly affected by the order in which participants were entered into the hyperalignment parameter calculations. We conducted an equivalent test with our data and found no significant impact on performance. This discrepancy might be attributed to increased variability between individuals in imagined vs. physical motor tasks as well as a difference in sample size between our study (N = 17) and Al-Wasity’s^41^ (N = 10). If participants have higher variability, the hyperalignment process may place unequal weight on the first individuals who set the initial reference point for alignment as well as the final participants whose data contributes more to the overall common model. If participants have higher inter-subject variability, the common model may be more influenced by subject order if an outlier enters alignment earlier or later in the hyperalignment process. Participants in our study demonstrated highly differentiable activity patterns (94.7% classification) for each of their executed finger movements. Perhaps in situations where the brain patterns are less well classified (e.g., in Al-Wasity’s^41^ study showing 48.3% classification of imagined movements), hyperalignment may be more sensitive to subject order and would benefit from larger pools of data.

Al-Wasity et al.^41^ also saw that aligning a new participant to a pre-established common model suffered a 12.5% drop in classification accuracy compared to using all individuals to inform the common model space. We conducted a similar analysis and saw no significant drop in prediction accuracy between these two approaches. Once again, this difference in findings could be due to less variability in activity patterns during motor execution versus imagery. Furthermore, our pre-established common model was informed by data from 16 other participants while Al-Wasity’s had only nine. Further research is warranted on hyperalignment between participants performing motor tasks vs. mental imagery to identify to precise cause of these discrepancies. Finally, we investigated how much data is needed to properly create a common model space or to align a new participant to a pre-established common model. Our results indicated that only two (of eight) runs of data (40 trials) are necessary to obtain an accurate alignment to a pre-established template.

One limitation of our functionally aligned neural templates of finger presses is that they would only be effective in participants with mild to moderate impairment. If unique activity patterns are not identifiable in at least two of the fingers, an accurate template would likely not be generated. Additionally, all analyses in this study were performed on data from healthy young adults. It remains untested if hyperalignment provides similar results between different age groups or impairment levels. Future work will need to explore similar approaches in older adults and individuals with mild fine motor impairments.

In conclusion, we introduce here a common model of individual finger presses that can be used as a surrogate training target during fMRI neurofeedback for participants with motor impairments. This neural template was derived from information from healthy young adults with high individuation abilities. Using such a model can benefit new participants by compensating for impaired digits, providing a larger pool of data to boost classification accuracy, and providing a clearer goal of neural activity to work towards.

## Methods

### Participants

Data from N = 17 participants recruited for previous studies^35,44^ was used in this experiment (6 female, all right-handed, average age 25.5 years, SD = 4.2). All participants provided informed consent before participation in this study. The Institutional Review Board at the University of Texas at Austin provided ethical approval. All methods were performed in accordance with the relevant guidelines and regulations of the University of Texas at Austin Institutional Review Board.

### Apparatus

A custom-built, MRI compatible keyboard was developed to collect the finger presses of the right index, middle, ring, and little fingers. The keyboard was equipped with four piano-like keys, each incorporating a sensor (Honeywell FSS015) to record the isometric force of each press at 120 Hz. Inside the scanner, the keyboard was secured to a wooden board which was placed in the participant’s lap. Participants received visual instructions and feedback via a back-projection screen presented using Python and Pygame.

### Imaging parameters

Participants were scanned in a Siemens Skyra 3 T scanner with a 32-channel head coil. The following EPI sequence was used for all individuals: TR = 2 s; 36 slices; in-plane resolution 2.3 × 2.3 mm; 100 × 100 matrix size; 2.15 mm slice thickness; 0.15 mm slice gap; 2 × multiband factor. A manual adjustment was performed after auto-alignment to the AC-PC plane to ensure adequate coverage of the sensorimotor cortex. A high-resolution anatomical scan (MEMPRAGE, 1 mm isotropic voxels) was also acquired to identify the primary sensorimotor cortex using Freesurfer.

### Region of interest

The regions of interest (ROIs) were defined as the primary motor (M1) and primary somatosensory (S1) cortices (Brodmann areas 4A, 4P, 3A, and 3B) and were identified in each participant using Freesurfer. These areas were selected as individuation of finger movements receives contributions from the corticospinal tract (CST) whose origins lie primarily in the M1 region^51–53^. This area produces low levels of force to control skilled movements^54^. Finger strength on the other hand receives input through the reticulospinal tract (RST) which has more distributed origins^55^. Furthermore, there is ample evidence of the connection between the M1 and S1 regions during motor tasks^56–59^. For the representational similarity analysis, the region of interest was further restricted to the hand knob area of the primary somatosensory cortex as described in Oblak et al.^35^.

### Data collection

Each participant underwent one task familiarization session outside the MRI and one localizer session inside the MRI scanner to identify unique representations in the sensorimotor cortex corresponding to each of the four fingers (index, middle, ring, and little). During the familiarization session, participants were allowed to practice the individual finger pressing task presented in Fig. 2 for 15 min. Each trial of the task consisted of 2 s to cue the target finger, 10 s of individual finger pressing, 1 s of rest, 2 s of feedback, and 1 s of rest before the next trial. The localizer session consisted of 8 runs of 20 trials each, lasting about 1 h.

Participants were instructed to hit the yellow targets with the cued finger while maintaining a constant force with the other fingers. One point was awarded for each target hit in this manner. No points were awarded if the non-cued fingers were outside the constant force cylinder, the participant did not remain within the target cylinder for 200 ms, or if the cued finger force exceeded the target force during a press. These measures ensured the participant was only pressing the target finger with an appropriate amount of force and speed. During each trial, a score bar was presented at the top of the screen along with a timer bar at the bottom. The score bar decreased in size as targets were hit up to a score of 15. After 15 points were acquired, the bar turned green and expanded in size.

After 10 s of finger pressing, a feedback thermometer was presented indicating the number of targets hit during the trial. The thermometer cylinder was colored green if 15 or more targets were hit or gray if less than 15 targets were hit. The initial maximum height of the feedback thermometer was equivalent to 15 points. This height was adjusted for each finger as participants increased their maximum score. The numerical score for the trial was also presented below the feedback thermometer. At the end of each run, the total score for all trials were displayed on the screen. For further details on experimental design or stimulus presentation, refer to Oblak et al.^35,44^.

### Within-subject decoding of individual finger presses

The fMRI data from the localizer session was used to train a classification model for individual finger presses. Standard preprocessing (rigid body motion correction, z-scoring, and detrending) was performed. The preprocessed fMRI data from the last 6 s of the pressing period was averaged to account for the hemodynamic delay resulting in a total of 160 time-averaged trials, 40 per finger. An ANOVA-based feature selection was also implemented to identify the top 100 voxels with highest variability. A support vector machine (SVM; scikit-learn) classifier was trained using a leave-one-run-out cross-validation.

### Between-subject decoding of individual finger presses using anatomical alignment

Individual participants’ functional data was transformed to the MNI152 (Montreal Neurological Institute) standard-space using FSL. Linear registration of each participant’s functional data to structural data was carried out using FSL’s FLIRT function. Nonlinear registration of structural data to the MNI template was implemented with FSL’s FNIRT function. Finally, all functional data was transformed to MNI space using applywarp. A new mask for sensorimotor ROI was also generated in the MNI space to be used by all participants. Data preprocessing was carried out in a similar manner as the within-subject analysis; however, a sensitivity analysis on the number of features used during classification revealed that including all voxels from the new sensorimotor mask yielded the best results. An SVM classifier was then trained using a leave-one-subject-out cross-validation approach.

### Between-subject decoding of individual finger presses using hyperalignment

Hyperalignment transformation was carried out in three stages^42^. In the first stage, data from two participants were brought into optimal alignment using the Procrustean transformation. Then, a third individual was brought into alignment with the mean response from the first two participants. This process was continued for each remaining individual by iteratively aligning them to the mean responses of the previous participants. In the second stage, this iterative process was repeated to align each individual with the group mean response from the first stage. The third stage consisted of calculating the transformation parameters necessary to bring each participant into alignment with the common model space generated in stage two.

Data preprocessing and feature selection was carried out prior to hyperalignment as described previously. The common model space was generated from all participants using the seven runs left out during cross-validation. The SVM classifier was then trained on these seven runs from all but one left out subject (leave-one-subject-out) and tested on the one run not used for feature selection or hyperalignment calculation for the left-out subject (leave-one-run-out). This was to ensure that the model was only trained on data from other participants and that there was no peeking between data in the training and testing set.

### Calculating mean deviation of uninstructed fingers

The enslavement of the uninstructed fingers was calculated by finding the mean deviation of force from baseline in accordance with Xu et al.^49^. For each press ($$p)$$ in a single trial $$(t=10s)$$, the mean deviation of force from each uninstructed finger $$({F}_{p,j})$$ from that finger’s baseline force $$({BF}_{j})$$ was calculated. This was averaged over all trials $$(T)$$ for each target finger:

1 $$meanDevP=\frac{1}{T}\sum_{t=1}^{T}\sqrt{\sum_{j=passive}{({F}_{p,j}-{BF}_{j})}^{2}}$$

where $j$ signifies the $j$th uninstructed finger.

During each press of the target finger, participants were instructed to keep the uninstructed fingers within a force range of 0.2–1.5 N. The baseline force for each uninstructed finger during the pressing of any given target finger was obtained by taking the mean force of the uninstructed finger when the target finger press was released (force < 1.5 N). The enslaved deviation force was then obtained for the uninstructed finger by averaging the mean force of that finger when the target finger was pressed (force > 2 N) subtracted by that finger’s baseline force.

### Representational similarity analysis

Representational similarity of the individual finger activity patterns was obtained by calculating the Pearson’s correlation (*r*) between the averaged fMRI data of each finger^50^. The dissimilarity was then found by subtracting *r* from unity. The dissimilarity matrices were calculated for each participant and averaged to provide the mean dissimilarity presented in Fig. 4a. Multidimensional scaling was also applied to project the representational structure into a two-dimensional plot.

### Removing fingers from hyperalignment parameter calculations

Data from one, two, or three fingers was removed from the dataset before feature selection and creation of the common model. Feature selection and hyperalignment calculations were conducted on the remaining fingers and applied to the dataset including all fingers. The SVM classifier was then trained and tested on this dataset in a leave-one-subject-out cross-validation. This process of data removal was repeated for each finger combination. The purpose of this process was to completely prevent information from the removed fingers from being used to inform the common model. This enforced the model to use only the data from the remaining fingers and other participants to infer the representations of the removed digits.

### Aligning a new participant to a pre-established common model

To create this common model space, we performed feature selection and hyperalignment as described above on all runs from *N*-1 participants. Then, the left-out individual was brought into alignment with this template using a leave-one-run-out cross-validation for feature selection and hyperalignment parameter calculations. An SVM classifier was trained on the data from the 16 participants used to create the template and tested on the left-out individual following each cross-validation fold.

### Varying amount of training data

To assess how much data is required to accurately hyperalign individuals and train a classifier, we reconducted our classification analyses using varying amounts of training data. We conducted a leave-one-run-out cross-validated analysis to hyperalign and train each classifier as described above starting with only two runs of data for each participant. One additional run was added after each calculation up to eight total included runs.

### Statistical analysis

A one-way analysis of variance (ANOVA) was run to compare classification accuracies between the hyperaligned, anatomically aligned, and within-subject models. A similar ANOVA test was also run to compare classification accuracies between groups when select fingers were removed during hyperalignment. A Welch F-test was performed due to the violation of the homogeneity of variance assumption in both cases. A Games-Howell post hoc test was conducted to elucidate the differences between groups. A two-sided paired t-test was used to compare difference in prediction accuracy between groups using all participants to create the common model space and aligning a new individual to a pre-established common model space. A one-way repeated ANOVA with Greenhouse–Geisser correction was used to identify improvements in classification accuracy as training data is increased. Tukey post hoc tests were utilized to identify how many runs were needed accurately align individuals and train a classifier.

## Data Availability

The datasets analyzed during the current study are available from the corresponding author on reasonable request.
